# Sulfur bacteria in wastewater stabilization ponds periodically affected by the ‘red-water’ phenomenon

**DOI:** 10.1007/s00253-012-3931-5

**Published:** 2012-02-23

**Authors:** Abdelaziz Belila, Ben Abbas, Imed Fazaa, Neila Saidi, Mejdi Snoussi, Abdennaceur Hassen, Gerard Muyzer

**Affiliations:** 1 Water Treatment and Reuse Laboratory, Water Researches and Technologies Centre of Bordj-Cedria, BP. 273, 8020 Soliman, Tunisia; 2 Department of Biotechnology, Delft University of Technology, NL-2628 BC Delft, The Netherlands; 3 Department of Aquatic Microbiology, Institute for Biodiversity and Ecosystems Dynamics, University of Amsterdam, NL-1098 XH Amsterdam, The Netherlands

**Keywords:** Sulfur bacteria, PCR–DGGE, Functional diversity, Red-water phenomenon, Wastewater stabilization ponds

## Abstract

Several wastewater stabilization ponds (WSP) in Tunisia suffer periodically from the ‘red-water’ phenomenon due to blooming of purple sulfur bacteria, indicating that sulfur cycle is one of the main element cycles in these ponds. In this study, we investigated the microbial diversity of the El Menzeh WSP and focused in particular on the different functional groups of sulfur bacteria. For this purpose, we used denaturing gradient gel electrophoresis of PCR-amplified fragments of the 16S rRNA gene and of different functional genes involved in microbial sulfur metabolism (*dsr*B, *apr*A, and *puf*M). Analyses of the 16S rRNA revealed a relatively high microbial diversity where *Proteobacteria*, *Chlorobi*, *Bacteroidetes*, and *Cyanobacteria* constitute the major bacterial groups*.* The *dsr*B and *apr*A gene analysis revealed the presence of deltaproteobacterial sulfate-reducing bacteria (i.e., *Desulfobacter* and *Desulfobulbus*), while the analysis of 16S rRNA, *apr*A, and *puf*M genes assigned the sulfur-oxidizing bacteria community to the photosynthetic representatives belonging to the *Chlorobi* (green sulfur bacteria) and the *Proteobacteria* (purple sulfur and non sulfur bacteria) phyla. These results point on the diversity of the metabolic processes within this wastewater plant and/or the availability of sulfate and diverse electron donors.

**Electronic supplementary material:**

The online version of this article (doi:10.1007/s00253-012-3931-5) contains supplementary material, which is available to authorized users.

## Introduction

Wastewater stabilization ponds (WSPs) are an extremely effective, natural form of wastewater treatment. They combine simplicity, robustness, and low cost with a very high degree of purification. They rely upon the natural ability of a shallow water body to achieve self-purification, whereby light penetration is of fundamental importance (Curtis et al. 1994). WSPs are usually designed as one or several series of anoxic, facultative, and maturation ponds, with the first two being mainly responsible for the removal of suspended solids and organic matter (biological oxygen demand) and the last one for the removal of pathogens and nutrients. Their low operation and maintenance costs have made them a popular choice for wastewater treatment, particularly in developing countries, since there is little need for specialized skills to operate these systems. WSP systems are widely used in Mediterranean countries (Mara 2008); in Tunisia, they account for more than 25% of the wastewater treatment plants (ONAS 2009). However, although these systems are very effective in the treatment of wastewater, they sometimes suffer from severe problems, such as the ‘red-water’ phenomenon.

The ‘red-water’ phenomenon is a temporal change in water color resulting from the massive growth (‘blooming’) of phototrophic anoxygenic purple bacteria. This bacterial phenomenon has been observed in several different wastewater stabilization ponds (Veenstra et al. 1995). The ‘red-water’ phenomenon is a sign of process failure causing malfunction of the wastewater treatment (Belila et al. 2009). The phenomenon is caused when the WSP system is overloaded with organic material, which stimulates sulfate reduction in the anoxic and facultative ponds. Consequently, the rising sulfide concentration is toxic for the algae but stimulates the growth of phototrophic sulfur bacteria that flourish under these anoxic conditions (Villanueva et al. 1994). The red-water phenomenon causes a deterioration of the effluent quality, i.e., red-colored water, a strong hydrogen sulfide smell, and high concentrations of suspended solid (Nair 1992).

The sulfate-reducing and the sulfur-oxidizing bacteria (SOB) encompass phylogenetically and physiologically diverse groups. The first group fall into three major branches: the *delta*-subclass of *Proteobacteria*, the Gram-positive bacteria, and branches formed by the thermophilic *Archaeal* sulfate-reducing bacteria (SRB) (Tang et al. 2008), while the taxonomic affiliation of sulfur-oxidizing bacteria has a broad range, from α-, β-, γ-, ε-*proteobacteria*, and *Chlorobia* to *Chloroflexi* (Ghosh and Dam 2009; Vannini et al. 2008). Both bacterial guilds are of immense importance from the industrial and environmental points of view and thrive in a wide variety of natural and engineered ecosystems (Asano et al. 2007; Ben-Dov et al. 2007). Their ecological and biogeochemical importance was recognized early due to their key role in the nitrogen, carbon, and sulfur cycles (Madigan 1995).

The anoxygenic phototrophic bacteria (APB) are responsible for the oxidation of sulfide as a major biogeochemical activity in aquatic ecosystems (Pierson and Olson 1987) since most known groups of APB, such as the purple sulfur, the purple non-sulfur, the green sulfur, and green non-sulfur bacteria, are able to use reduced sulfur compounds as electron donors for anoxygenic photosynthesis (Dahl and Prange 2006; Sander and Dahl 2008).

A wide variety of molecular tools have been applied to assess the diversity of bacteria involved in the sulfur cycle by targeting the16S rRNA gene (Dar et al. 2007; Lücker et al. 2007). However, the lack of phylogenetic coherence among both bacterial guilds and the different metabolic pathways involved in the oxidative and reductive processes of the sulfur cycle limit the use of 16S rRNA genes for the detection and the ecophysiological assignment of these bacteria. Targeting functional genes implicated in the microbial sulfate reduction and sulfur oxidation processes is a better strategy to analyze the phylogenetic complexity of bacteria of the sulfur cycle, specifically the sulfate-reducing and the sulfur-oxidizing bacteria (Kubo et al. 2011; Miletto et al. 2007). Functional genes such as *dsr*B (Geets et al. 2006), *Sox* (Meyer et al. 2007), *apr*A (Meyer and Kuever 2008), and *puf*M (Ranchou-Peyruse et al. 2006) have been successfully applied to resolve the genetic diversity of both sulfate-reducing and sulfur-oxidizing bacteria.

The dissimilatory sulfite reductase (DSR) and adenosine-5′-phosphosulfate (APS) reductase are two keys enzymes in microbial sulfate reduction and sulfur oxidation processes, highly conserved among the sulfate-reducing and the sulfur-oxidizing bacteria, and consequently *dsr*AB and *apr*AB genes constitute the most suitable targets for molecular profiling of the microbial community structure of the sulfur cycle in the environment (Meyer and Kuever 2007a). In addition, the *puf*M gene encoding the M subunit of the photosynthetic reaction center in purple sulfur and purple non-sulfur bacteria (Corson et al. 1999) has been successfully applied to the phylogenetic characterization of phototrophic bacterial communities in aquatic environments.

So far, most studies on WSP systems have concentrated on the fate and removal of pathogenic microorganisms, such as fecal enterococci, *Cryptosporidium*, *Giardia* (Anceno et al. 2007; Reinoso and Bécares 2008), and helminth eggs (e.g., *Taenia*, *Ascaris*, and *Trichuris*) (Tyagi et al. 2008). However, here we present for the first time a detailed study on the microbial communities present in WSP systems. We focused on the bacterial diversity during ‘red-water’ phenomenon occurrence and especially on the diversity of the sulfate-reducing and the sulfur-oxidizing bacteria.

## Materials and methods

### Wastewater stabilization ponds

Our study was carried out in a wastewater stabilization pond (WSP) system located in the city Mutuelleville in north-east of Tunisia (36°49′ N, 10°10′ E). The WSP system consists of four inter-connected ponds: an anaerobic, a facultative, and two maturation ponds. The geometric characteristics of these ponds are summarized in Table 1. The system mainly receives wastewater of domestic origin, i.e., ‘black’ water (water from toilets) and ‘grey’ water (domestic sewage). The wastewater fills up the first, anoxic (A) pond, then enters the secondary facultative (F) pond through an outflow, and subsequently enters the maturation (M) pond. Finally, the treated water is released in a small river.

**Table 1:** Geometric characteristics of the wastewater stabilization ponds

	Anaerobic pond	Facultative pond	Maturation pond
Surface (m²)	30	100	122
Depth (m)	3.5	2.34–1.44	1.34
Volume (m^3^)	96	180	164
Water depth (m)	3.3	2	1.15

### Environmental sampling

For molecular analysis, 16 samples were collected in April 2009. Five sediment samples each were collected from the anaerobic (S_A1_ to S_A5_) and from the facultative pond (S_F1_ to S_F5_) with a Plexiglas core tube (5 cm in diameter and 20 cm in length). Sediment samples were taken from the middle and each corner of the basins. The sediment layers within the maturation ponds were very thin and could not be sampled. Six water samples (W_A_, W_F_, W_M1_, W_M2_, W_M3_, and W_M4_) were collected and pre-filtered through polycarbonate filters (0.8 μm in pore size) to exclude debris.

### Physical and chemical parameters

The in situ temperature and pH were measured with a handheld pH and temperature meter (WTW 340i model, WTW, Weilheim, Germany). The dissolved oxygen (DO) concentration was determined using a Multiline F/set P4 universal meter (WTW, Weilheim, Germany). The 5-day biochemical oxygen demand (BOD_5_), chemical oxygen demand (COD), and total suspended solids (TSS) as well as the sulfate and sulfide concentrations were determined according to the analytical methods described in “Standard Methods for the Examination of Water and Wastewater” (APHA 1995). Chlorophyll *a* concentration was estimated by the methanol extraction method (Pearson 1986).

### Nucleic acid extraction

Prior to DNA extraction, water samples (250 ml) were centrifuged at 13,500 rpm at 4 °C. A total of 0.5 g of concentrated biomass and the same quantity of sediments were used for DNA extraction by using the UltraClean Soil DNA Extraction Kit (MOBIO Laboratories, Inc., CA, USA) according to the manufacturer’s instructions.

### PCR amplification

Amplification of 16S rRNA and *dsr*B gene fragments was performed using the primer pairs 341F-GC and 907R (Muyzer et al. 1995) and DSRp2060F-GC and DSR4R (Wagner et al. 1998), respectively. For the amplification of the *dsr*B gene, a “touchdown” protocol was used, wherein the annealing temperature was decreased from 65 to 55 °C in 20 cycles. Thermal cycling was carried out as follows: 5 min of initial denaturation at 95 °C, followed by 20 cycles of denaturation at 95 °C for 40 s, a “touchdown” annealing step for 40 s, and elongation at 72 °C for 1 min. This was followed by another 30 cycles of denaturation at 95 °C for 40 s, annealing at 55 °C for 40 s, and elongation at 72 °C for 1 min (Dar et al. 2007). Amplification was completed by a final elongation step at 72 °C for 10 min. DNA from *Desulfobulbus propionicus* was used as a positive control and deionized water as a negative control in all PCR amplifications. A ‘touchdown’ PCR protocol was used to amplify *apr*A gene fragments using primers AprA-1-FW and AprA-5-RV-GC (Meyer and Kuever 2007c). Thermal cycling was carried out as follows: 5 min of initial denaturation of DNA at 95 °C, followed by 35 cycles of denaturation at 95 °C for 60 s, a ‘touchdown’ annealing step for 90 s (annealing temperature was decreased in the first 20 cycles by 0.5 °C until reaching 50 °C in every cycle, while the subsequent 15 cycles were carried out at constant temperature), and elongation at 72 °C for 120 s. Amplification was completed by a final elongation step at 72 °C for 10 min. Primer set *puf*M557F and *puf*M750R was used to amplify the photosynthetic unit-forming gene (*puf*M) of purple phototrophic bacteria (Achenbach et al. 2001). PCR was performed by using an initial denaturation step at 95 °C for 15 s, followed by 35 amplification cycles of denaturation (95 °C for 1 min), annealing (54 °C for 30 s), elongation (72 °C for 1 min), and a final extension step at 72 °C for 10 min. Amplifications were performed in Biometra T Gradient Thermocycler (Biometra GmbH, Germany), and PCR products were verified on 1% or 2% (*w*/*v*) agarose gel in 1× Tris/acetate/EDTA (TAE) buffer.

### Denaturing gradient gel electrophoresis

All DGGE experiments were performed with the D-Code system (Bio-Rad Laboratories, CA, USA). For the 16S rRNA, electrophoresis was performed with 6% polyacrylamide gels (ratio of acrylamide to bisacrylamide, 40:1) submerged in 1× TAE buffer (40 mM Tris, 40 mM acetic acid, 1 mM EDTA, pH 7.5) at a constant temperature of 60 °C. The electrophoresis conditions for gene fragments were: 16 h at 100 V in a linear 20% to 80% denaturant gradient (100% denaturant was a mixture of 7 M urea and 40% [*v*/*v*] formamide) (Schäfer and Muyzer 2001). However, for *dsr*B gene fragments, a gradient of 35–80% denaturant (the 100% [*w*/*v*] denaturing solution contained 7 M urea and 40% [*v*/*v*] formamide) was constructed in a 1.5-mm-thick 8% polyacrylamide gel. The gel was initially run at 150 V for 5 min to facilitate the access of PCR products into the gel and then at a constant voltage of 75 V for 30 h in a 0.5× TAE buffer at a stable temperature of 60 °C (Miletto et al. 2007). For *puf*M gene fragments, samples were electrophoresed on a 10% polyacrylamide gel with 20% to 80% denaturant (100% denaturant was 7 M urea and 40% [*v*/*v*] formamide) at 130 V for 8 h at 60 °C (Karr et al. 2003). For *apr*A, a double-gradient DGGE was used with a linear polyacrylamide gradient of 6–8% and a linear denaturant gradient of 30–60% (100% denaturant was 7 M urea and 40% [*v*/*v*] formamide. Electrophoresis was performed at 60 °C for 2 h at 150 V and subsequently for 2 h at 200 V. All DGGE gels were stained with SYBR Green I (Sigma-Aldrich Corporation, St. Louis, MO, USA) and were visualized on a UV transilluminater. Individual bands were excised, re-suspended in 20 μl of Milli-Q water, and stored overnight at 4 °C. A volume of 3 to 5 μl of the supernatant was used for re-amplification using the original PCR conditions and primer pair without a GC clamp and then photographed using a G:BoxiChemi 2D gel image analyzer using Genesnap software 7.03 (SYNGENE, Synoptics, Ltd, Cambridge, UK).

### Comparative sequence analysis

Sequences obtained from the excised DGGE bands were first compared to sequences stored in the publicly accessible database GenBank using the NCBI BLAST search tool (http://blast.ncbi.nlm.nih.gov/Blast.cgi). Subsequently, the sequences were imported into the ARB software package (Ludwig et al. 2004) and aligned using the automatic aligner function. The phylogenetic trees were constructed on the basis of long (more than 1,300 bp) sequences using different methods integrated within the ARB software. Partial sequences obtained in this study were then inserted into the pre-established tree using the ARB parsimony tool.

### GenBank accession numbers

The nucleotide sequence data are available under the GenBank accession numbers, HQ222639 to HQ222674 (*dsrB*), HQ222675 to HQ222729 (*pufM*), HQ222613 to HQ222638 (*aprA*), and HQ222730 to HQ222810 (16S rDNA).

## Results

### Performance of the wastewater stabilization pond system

The performances of the wastewater stabilization pond system investigated in April 2009 were unsatisfactory regarding to the BOD and COD removal efficiency (Table 2). The chemical and biological parameters within the stabilization ponds gave evidence of a eutrophic state. The system performance decreased during this period of the year and the percentage removal of the TSS as well as the biological and chemical oxygen demands (BOD_5_ and COD) were unsatisfactory (50%, 40%, and 43%, respectively). The proliferation of the purple red color throughout the water column, reaching up to the surface of the four ponds, supports the prevalence of anoxic conditions within the whole system. Wastewater treatment successfully enhanced SO_4_^2−^ removal, accounting for greater than 83% reduction in SO_4_^2−^ concentrations during the sampling campaign. Maximum sulfate removal (86%) was reached within the first maturation pond (M1), showing a distinctive development of sulfates reducing conditions within this pond.

**Table 2:** Physical and chemical parameters of the wastewater stabilization pond system

	*T* (°C)	pH	DO (mg/L)	TSS (mg/L)	BOD_5_ (mg/L)	COD (mg/L)	Chl a (μg/L)	SO_4_^2-^ (mg/L)	S^2-^ (mg/L)	Salinity (mg/L)
A	14.7 ± 2	6.9 ± 0.2	0.20 ± 0.2	533 ± 18	473 ± 19.3	908 ± 13	156 ± 28	320 ± 33	40.8 ± 0.7	2 ± 0.4
F	13.5 ± 1	7.6 ± 0.2	2.50 ± 0.4	342 ± 38	360 ± 19.6	720 ± 17	2,634 ± 268	245 ± 43	25 ± 0.9	1.4 ± 0.6
M1	12.2 ± 2	7.5 ± 0.1	3.10 ± 0.3	247 ± 18	283.3 ± 11.6	510 ± 26	3,456 ± 265	45 ± 15	9 ± 3.2	1.1 ± 0.5
M2	12.9 ± 2	7.4 ± 0.1	3.10 ± 0.9	262 ± 0.9	262 ± 18	505 ± 11	3,288 ± 345	52 ± 17	10 ± 0.7	1.1 ± 0.3

*A* anaerobic ponds, *F* facultative pond, *M1* maturation pond 1, *M2* maturation pond 2

### DGGE analysis of 16S rRNA gene fragments

DGGE analysis of PCR-amplified 16S rRNA gene fragments showed many different bands, between 12 and 23 per lane, indicating a high microbial diversity in the WSP system (figures of the DGGE gels are provided as “Electronic supplementary material”). Largely reproducible patterns were obtained with the five sediment samples from the anaerobic pond (lanes 1–5) and the five samples from the facultative pond (lanes 6–10). Differences within the DGGE profiles appeared more pronounced among the water samples (lanes 11–16) and between the water and the sediment samples than among the sediment samples (lanes 1–10). Several bands were detected concomitantly within different samples, although with varying intensities (i.e., bands A_2_, G_2_, H_2_, C_2_, and D_2_). In total, 90 bands were excised from the denaturing gel and sequenced in order to identify the predominant community members. A total of 15 DGGE bands gave ambiguous sequences and were not included in the phylogenetic analysis.

**Fig. 1:**
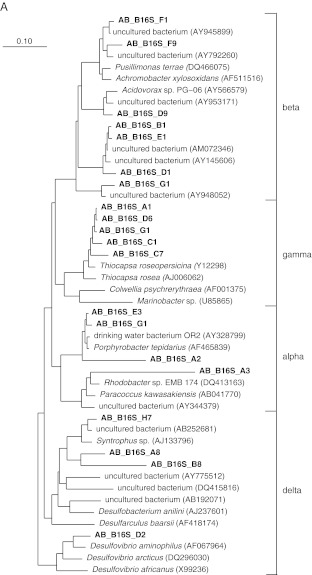

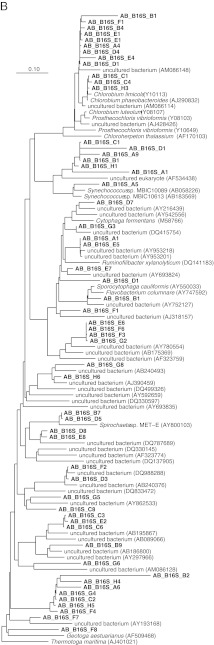

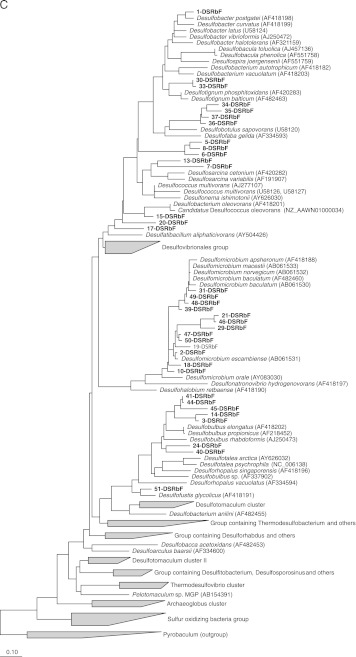

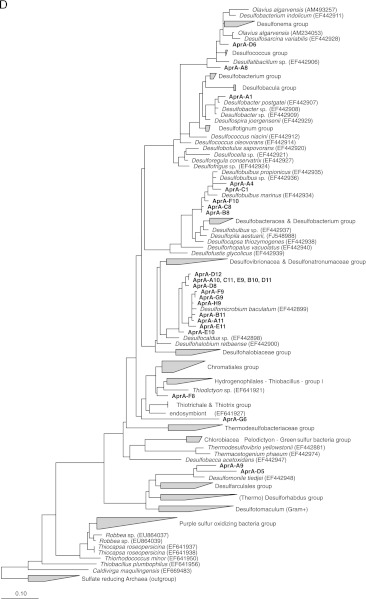

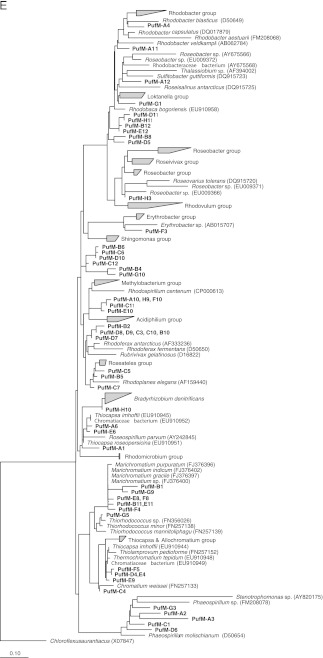
Phylogenetic trees based on sequences of the 16S rRNA *Proteobacteria* (**a**), 16S rRNA, non-*Proteobacteria* (**b**), dsrB (**c**), aprA (**d**), and pufM (**e**). For the *dsr*B gene phylogenetic tree: the ‘base’ tree was calculated using neighbor joining algorithm on the ~1,500 positions within 1F–4R primer region of the *dsr*AB gene. PAM protein correction was used together with a filter (ignoring the third base pair). The sequences derived from the DGGE gel were added, after (manual) correction, using ARB parsimony (quick add species to existing tree). For calculation, 360 positions were used also applying a filter (ignoring the third base pair). For the *apr*A gene phylogenetic tree: the ‘base’ tree was calculated using neighbor joining algorithm on the ~1,109 positions of long sequences within the AprA gene. Felsenstein correction was used together with a filter (ignoring the 3rd base pair). The sequences derived from the DGGE gel were added, after (manual) correction, using ARB parsimony (quick add species to existing tree). For calculation, maximum 242 positions were used also applying a filter (ignoring the third base pair). Bootstrap (1,000×) analysis was performed and values were written in the tree. For the *puf*M gene phylogenetic tree: the ‘base’ tree was calculated using neighbor joining algorithm on the ~632 positions of long sequences within the *puf*M gene. Felsenstein correction was used together with a filter (ignoring the third base pair). The sequences derived from the DGGE gel were added, after (manual) correction, using ARB parsimony (quick add species to existing tree). For calculation, a maximum of 518 positions (or as many as were available) were used also applying a filter (ignoring the third base pair)

The phylogenetic affiliation of the different community members present in the WSP system is presented in Fig. 1a, b. A total of 26 sequences (28.8%) were attributed to different classes within the *Proteobacteria* (Fig. 1a), i.e., the *Betaproteobacteria*, the *Gamma*-, the *Alpha*-, and the *Deltaproteobacteria*. The other 64 sequences showed similarity with sequences from other phyla; 14 sequences (15.5%) grouped with sequences in the phylum *Chlorobi*, 18 other sequences (20%) with the phylum *Bacteriodetes*, 13 (14.4%) with the phylum *Cyanobacteria,* 4 (4.4%) with *Bacteroidetes*/*Chlorobi* group, 4 (4.4%) with the phylum *Spirochaeta*, 3 (3.3%) with the phylum *Synergistetes*, 4 (4.4%) with the phylum Acidobacteria, 1 (1%) with the phylum *Thermotoga* , and 3 (3.3%) with uncultured bacterium partial 16S rRNA gene.

The sequences corresponding to bands C_6_, C_3_, and E_2_ showed 99% and 100% similarity with uncultured *Aminanaerobia* bacterium 16S rRNA gene clones. The closest relative for DGGE band sequences F_2_ and D_3_ was uncultured *Acidobacteria* bacterium 16S rRNA gene with 99% similarity. Sequences of bands B_10_, C_10_, and A_5_ had 93% and 95% similarity with uncultured cyanobacterium clone 16S ribosomal RNA gene and those related to bands F_3_, G_2_, E_6_, and F_6_ showed 99% similarity with uncultured *Bacteroidetes*/*Chlorobi* group bacterium clone. The closest relatives for bands D_11_ and B_12_ was an uncultured *Burkholderiaceae* bacterium with similarity percentage of 95% and 99%, respectivelty. DGGE band E_4_ was closely related to an uncultured *Chlorobiaceae* bacterium with 99% similarity while DGGE band sequence C_12_ showed 93% similarity with an uncultured *Chromatiaceae*. Band F_8_ sequence was closely related to uncultured *Bacteroidetes* bacterium 16S rRNA gene with 99% similarity. Notably, most of the 16S rRNA gene sequences were affiliated to uncluttered bacteria (Fig. 1a, b); thus, the use of this phylogenetic marker could not conclude on the physiological properties and specific biochemical pathways acting in the targeted communities.

Bacteria involved in the sulfur cycle identified within the WSP were represented by the SRB of the *Deltaproteobacteria* (*Desulfovibrio* spp.), the gammaproteobacterial purple sulfur bacteria (PSB) (*Thiocapsa* spp.), the alphaproteobacterial purple non-sulfur bacteria (PNSB) (*Rhodobacter* spp.), and the green sulfur bacteria (GSB) (*Chlorobium* spp.). The sequence of band D_2_ was closely related (96% similarity) to a *Desulfovibrio* sp. Sequences corresponding to bands C_7_ and D_6_ had 95% and 99% similarity with a sequence of *Thiocapsa* sp., respectively. The closest relative for bands A_11_ and G_12_ was *Thiocapsa pendens* (99% similarity). The sequence of band C_12_ showed 93% similarity to the sequence of an uncultured bacterium within *Chromatiaceae.* Members belonging to the green sulfur bacteria were, however, the most prominent group within the WSP with 11 sequences. Sequences of bands C_1_ and H_3_ had 100% and 99% similarity with the 16S rRNA sequence of *Chlorobium phaeobacteroides*, respectively, while the sequence of band C_4_ had 98% similarity with the sequence of *Chlorobium limicola*. The sequences of band E_4_ showed 99% similarity to the sequence of an uncultured bacterium within the *Chlorobiaceae*.

### DGGE analysis of *dsr*B gene fragments

DGGE analysis of the *dsr*B gene fragments showed the highest diversity in sediments of the anaerobic and facultative ponds (“Electronic supplementary material”). Some of the DGGE bands (i.e., bands 1 and 2) were detected only in the sediments and not in the water phase. The SRB community composition varied significantly within all sediment samples as shown in “Electronic supplementary material”, except that DGGE bands 5 and 6 were detected in all anaerobic pond sediment samples. The SRB community diversity decreased within the sediment of the facultative pond (lanes 9 and 10, “Electronic supplementary material”) and then increased in the maturation pond water samples (lanes 15 and 16, “Electronic supplementary material”). The number of bands per lane varied between 4 (lane 11) and 25 (lane 7).

A total of 36 bands were sequenced and used for phylogenetic analysis. All sequences were assigned to sulfate-reducing bacteria within the *Deltaproteobacteria*. Band 48 had a 97% similarity to the *dsr* sequence of *Desulfomicrobium macestii*. The closest relative for band 1 was *Desulfobacter postgatei* (93% similarity). The sequence corresponding to band 30 showed 87% similarity to *Desulfotignum balticum*. Five *dsr*B sequences were assigned to the *Desulfobulbus* genus with similarity levels varying from 84% to 90%. DsrB sequences of bands 40, 41, and 44 had 84% and 90% similarity with *D. propionicus*, respectively, while sequence of band 45 had 87% similarity with *Desulfobulbus rhabdoformis*. The sequence corresponding to band 24 had a similarity of 87% with uncultured *Desulfobulbus* sp. The sequences corresponding to bands 2, 19, 46, and 50 were affiliated to members of the genus *Desulfomicrobium*. The closest relative for bands 2 and 46 was *Desulfomicrobium escambiense* with 97% similarity. Band 19 had 93% similarity with *Desulfomicrobium* sp., while band 50 showed 97% level of similarity with *Desulfomicrobium* sp. ADR28. Several *dsr*B-related sequences were assigned to uncultured sulfate-reducing bacteria, such as band 3 and 14, which had 99% and 97% levels of similarity with *dsr*B gene sequences of uncultured sulfate-reducing bacteria, respectively. The sequence corresponding to band 15 had a similarity of 94% with *Desulfococcus oleovorans* Hxd3. The sequences corresponding to bands 34, 35, 36, and 37 were affiliated to *Desulfobotulus sapovorans*, a Gram-negative fatty acid-oxidizing species (Devereux et al. 1989) which is a member of the *Desulfobacteraceae* family. The *dsr*B sequences of DGGE bands 5, 6, and 8 were affiliated with an uncultured sulfate-reducing bacterium clone LGWI06 with a percentage similarity of 84%, whereas DGGE bands 17 and 20 sequences were affiliated with *Desulfatibacillum aliphaticivorans*, an *n*-alkane- and *n*-alkene-degrading, sulfate-reducing bacterium (Cravo-Laureau et al. 2004). The *dsr*B sequences of bands 7 and 13 were assigned to the *Desulfosarcina*/*Desulfococcus* cluster. The dsrB band 51 showed 94% similarity with *Desulfofustis glycolicus*. No Gram-positive spore-forming SRB were detected.

### DGGE analysis of *apr*A gene fragments

Because sulfate-reducing as well as sulfur-oxidizing prokaryotes use APS reductase, the genes of this reversible enzyme can be found in both groups; the use of the *apr*A gene allowed the concomitant identification of both SRB and photosynthetic SOB within the WSP. Twenty-four of the sequenced *apr*A bands were affiliated with sulfate-reducing bacteria belonging to the *Deltaproteobacteria*. Representatives of the sulfate-reducing bacteria community indeed were assigned to the *Desulfobacteraceae* family. The sequence of band A_1_ had a high similarity of 99% with the *apr*A sequence of *D. postgatei.* The closest relative for the sequences D_6_ and A_8_ was *Desulfonema ishimotonii* (95%). The sequences corresponding to DGGE bands A_4_, F_10_, C_1_, B_8_, and C_8_ belonged to representatives of the *Desulfobulbus* genus. Sequence analysis showed that bands A_4_, B_8_, and F_10_ had 95% similarity with *Desulfobulbus* sp. DSM 2033, while band C sequence had 89% similarity with *D. propionicus*. The closest relatives for bands A_9_ and D_5_ were *Syntrophobacter wolinii* (87%) and *Desulfomonile tiedjei* (93%), respectively (*Syntrophaceae* family)*.* Most of the analyzed *apr*A gene sequences (58%) were affiliated with *Desulfomicrobium baculatum* with a similarity of 97% (DGGE bands A_11_ and G_9_). No spore-forming SRB neither sulfur-oxidizing bacteria *apr*A-related sequences were founded. Two *apr*A gene-related sequences (G_6_ and F_8_) were affiliated to *Gammaproteobacterial Thiodictyon*, a phototrophic sulfur-oxidizing bacterium of the *Chromatiaceae* family.

### DGGE analysis of *puf*M gene fragments

Phylogenetic analysis of the *puf*M sequences revealed the presence of members belonging to three different photosynthetic bacterial groups, the PSB, the PNSB, and the aerobic anoxygenic phototrophic bacteria (AAPs). A total of 17 *puf*M band sequences belonged to the purple sulfur bacteria and in particular to the family *Chromatiaceae.* The sequences corresponding to bands E_4_, B_10_, and D_7_ showed a 96% and 89% similarity to the sequence of *Thiorhodococcus drewsii*, respectively. DGGE bands C_1_ and D_8_ had a 90% and 88% sequences similarity with *Thiocystis violacea* and band A_1_ had a high level of similarity (99%) with *Allochromatium phaeobacterium*. The closest relative for A_6_ and E_6_ band sequences was *Allochromatium vinosum* (97% similarity). Five sequences were affiliated to members of genus *Thiocapsa* with different levels of similarity. The sequences corresponding to bands C_4_, C_7_, and F_5_ showed a 91%, 93%, and 95% similarity to *Thiocapsa* sp. MTV2IF083, respectively. Bands D_4_ and G_5_ sequences had 96% similarity with *Thiocapsa* sp. MTRDDF078 and *Thiocapsa* sp. MTWDM010, respectively. The closest relative to E_9_ sequence was the marine bacteria *Marichromatium indicum* (92% similarity).

The *Puf*M gene-related sequences of bands B_2_, D_8_, D_9_, C_3_, C_10_, B_10_, and D_7_ were clustered with members of the genus *Rubrivivax* and *Rhodoferax* of *Betaproteobacteria*. Sequences of bands (A_2_, A_3_, C_1_, G_3_, and D_6_) were affiliated to the phototrophic purple non-sulfur *Phaeospirillum* genus of the *Rhodospirillales* family, while those of bands (D_11_, H_11_, B_12_, E_12_, B_8_, and D_5_) were affiliated to the photosynthetic purple non-sulfur bacteria *Rhodobaca bogoriensis* of *Alphaproteobacteria.* The PNSB were represented also by three different families: *Bradyrhizobiaceae*, *Rhodobacteraceae*, and *Rhodospirillaceae*. Three DGGE bands, D_5_, A_4_, and A_11_, had similarities of 98%, 88%, and 87% with the sequences of *Rhodobacter sphaeroides* and *Rhodobacter blasticus*, respectively, while the closest relatives for the sequences of bands H_10_, A_2_, and G_1_ were *Rhodopseudomonas* sp*.* (88%), *Rhodospirillum centenum* (87%), and *Rhodobacteraceae* bacterium (90%), respectively.

Representatives of the aerobic photosynthetic bacteria were identified and were assigned to three different families within *Alphaproteobacteria*: *Erythrobacteraceae*, *Rhodobacteraceae*, and *Acetobacteraceae*. The sequence corresponding to band F_3_ showed 96% similarity to the sequence of *Erythromicrobium* sp. Sequences corresponding to bands H_3_ and B_8_ had similarities of 90% and 94% with *puf*M sequences of a *Roseobacter* sp. and *Roseococcus thiosulfatophilus*, respectively.

## Discussion

### Performance of the wastewater stabilization pond system

Wastewater stabilization ponds are without doubt the most important method of wastewater treatment in developing countries where sufficient space is available and where the temperature is most favorable for their operation. The deepest ponds (the anaerobic and facultative ponds) generally stratify between March and September due to their small size and as a result of warm climatic conditions and the absence of artificial aeration (Abis and Mara 2006). During this period, the sulfate reduction process becomes the dominant process in the sediments of these ponds and is stimulated by the increase in temperature. All ponds become fully anoxic and this was indicated by the black coloration of elemental sulfur deposits from the oxidation of hydrogen sulfide (Table 2). The stabilization ponds failure is reflected by the changes in the pond biology through the reduction of the wastewater treatment efficiency and the change of the WSP pigmentation. The hydrogen sulfide produced spontaneously reduces oxygen expanding the anaerobic zone throughout the water column within the facultative pond and may even diffuse into the maturation ponds. Sulfide toxicity affects algal growth (Konig et al. 1987) and stimulates the prevalence of anoxic conditions and the purple photosynthetic bacteria become visible at the surface (Lai and Lam 1997).

### Analysis of the total bacterial community

So far, most studies have focused on the removal of pathogens and only a few studies have focused on the characterization of microbial communities in wastewater stabilization ponds (Shammas et al. 2009). The major bacterial groups (i.e., *Proteobacteria*, *Chlorobi, Bacteroidetes*, and *Acidobacteria*) found in this study have been previously found in other wastewater treatment systems, such as activated sludge, aerated lagoons, and sewage treatment plants (Malik et al. 2008; Moura et al. 2009). The bacteria detected were related to known organisms involved in the degradation of diverse pollutants, suggesting a similar role of these microorganisms within the wastewater stabilization pond system. The 16S rRNA gene sequences affiliated with the *Proteobacteria*, *Chlorobi*, *Bacteroidetes*, and *Cyanobacteria* phyla were the most frequently retrieved. A similar bacterial community composition has been previously described from a wastewater treatment plant (Wagner et al. 1993; Wagner and Loy 2002). The predominance of the *Proteobacteria* was in accordance with previous results obtained by Boon et al. (2002) and Ding et al. (2010).

Representatives of the *Proteobacteria* classes were most abundantly present within the WSP; this result was in accordance with those obtained by Xia et al. (2010) who reported the predominance of the *Proteobacteria* within five biological wastewater treatment reactors. This bacterial phylum is known to flourish in eutrophic ponds and is responsible for the removal of the organic matters from municipal wastewater (Wagner et al. 2002). The distribution of the different classes of *Proteobacteria* varies according to the type of wastewater treated (Arroyo et al. 2010). In contrast, while the *Alphaproteobacteria* tend to dominate within activated sludge (LaPara et al. 2000), the proteobacterial community in our system was dominated by members of *Betaproteobacteria* and *Gammaproteobacteria*.

### SRB in wastewater treatment systems

SRB are present in wastewater treatment plants (Muyzer and Stams 2008) treating domestic (Baena et al. 1998) and industrial (Ben-Dov et al. 2007) wastewater. Sulfate reducers play a significant role in the anaerobic biomineralization pathways, especially in wastewater treatment systems (Oude Elferink et al. 1994) where sulfate reduction can be the dominant terminal electron-accepting process and can even account for up to 50% of mineralization of organic matter in wastewater.

16S rRNA, *apr*A-, and *dsr*B- genes analysis revealed the presence of the sulfate reducers within all water and sediment samples. The SRB community was phylogenetically diverse and all representatives are Gram-negative mesophilic SRB of the *Deltaproteobacteria* class. Representatives of this bacterial guild belonged to the family of *Desulfobacteraceae* (*Desulfobacter*, *Desulfotignum*, *Desulfobotulus*, and *Desulfococcus*), the family of *Desulfobulbaceae* (*Desulfobulbus* and *Desulfofustis*), the family of *Desulfomicrobiaceae (Desulfomicrobium*), the family of *Syntrophaceae* (*Desulfomonile*), and the family of *Syntrophobacteraceae* (*Syntrophobacter*). Most of these bacterial genera have been previously isolated and/or identified within wastewater treatment systems (Mohanakrishnan et al. 2011; Raskin et al. 1995). Similar sulfate-reducing bacteria community composition has been previously reported from wastewater environments, such as upflow anaerobic sludge bed wastewater treatment reactors (UASB) (Dar et al. 2005) or wastewater biofilm (Okabe et al. 1999).

The SRB are divided into two broad subdivisions that belie physiological and ecological roles of the two groups: complete and incomplete oxidizers. Complete oxidizers typically utilize a broader range of substrates than incomplete oxidizers and may be considered as generalists compared with the more specialist incomplete oxidizers. Representatives of both groups were identified within the WSP, complete oxidizers were represented by species of the genera *Desulfobacter*, *Desulfococcus*, *Desulfonema*, *Desulfosarcina*, and *Desulfomonile* (Rabus et al. 2006), while the incomplete oxidizers include representatives of the genera *Desulfovibrio*, *Desulfomicrobium*, and *Desulfobulbus* (Madigan and Martinko 2006). Incomplete oxidizers are present in the anoxic pond, while the complete oxidizers may be located in the following facultative and maturation ponds. Indeed the growth kinetics for incomplete oxidizers is generally faster than the complete oxidizers. However, the former are less versatile regarding their nutritional requirements (Widdel 1988); in addition, complete oxidizers have the ability to oxidize the organic compound to carbon dioxide, and incomplete oxidizers carry out the incomplete oxidation of the organic compound to acetate and CO_2_ which subsequently can be used by complete oxidizers further in the WSP system.

### Analysis of SOB-like sequences

Based on the 16S rRNA, *apr*A, and *puf*M gene analysis, the phylogenetic complexity of SOPs in the WSP consisted of putative strains of the GSB (*Chlorobi*), the *Gammaproteobacterial* PSB and the *Alphaproteobacterial* PNSB; all these bacteria have been shown to be able to oxidize reduced sulfur compounds (Brune 1995).

Many pufM sequences were related to the purple non-sulfur bacteria; these bacteria preferentially grow photoheterotrophically under anaerobic conditions in the light by using various organic substrates. Nevertheless and contrary to the misleading nomenclature, many of these bacteria are also able to use sulfur compounds as a source of electrons (Imhoff et al. 2005). It is now well established that a number of purple non-sulfur bacteria are able to grow photolithoautotrophically with reduced sulfur compounds such as *Rhodobacter*, *Rhodopseudomonas*, *Rhodoferax*, and *Rubrivivax*, which can use hydrogen, sulfide, thiosulfate, or ferrous iron as electron donors to support their anoxic, phototrophic growth (Kopriva et al 2008).

In contrast to results generated by the *puf*M DGGE-based approach, only two *apr*A sequences were affiliated to *Thiodictyon*, a sulfur-oxidizing bacterium which is an obligate and strictly anaerobic phototroph. This difference between the *puf*M- and the *apr*A-based approaches may be explained by the limited phylogenetic distribution of APS reductase-encoding genes among phototrophic bacteria, unlike the *sox* gene which is found in all *Chromatiaceae (*Meyer et al. 2007). Indeed *apr*AB gene distribution is restricted in *Chlorobiaceae* to members of sub-clusters 3 and 4b, to some species of *Chromatiaceae*, while absent throughout the *Rhodospirillaceae* and *Ectothiorhodospiraceae* families (Meyer and Kuever 2007b), thus limiting the utility of using *apr* genes to the survey of anoxygenic phototrophic SOB. The *puf*M gene universally distributed among all purple anoxygenic photosynthetic (Tank et al. 2009), which may explain the high diversity of the purple anoxygenic phototrophic bacteria revealed in comparison with the former genes (*dsr*B and *apr*A). This gene may constitute a better target to circumvent this limitation but this gene is not directly linked to the sulfide oxidation process and this may explain why many *puf*M-related sequences belonged to bacteria which were not able to use reduced sulfur compounds (i.e., PNSB). The primer set *puf*M557F-*puf*M750R used within this study allows the detection of both PSB and PNSB. It should be remembered that this primer set might also amplify *pufM* gene fragment of aerobic phototrophs (Shimada 1995) and the green non-sulfur bacterium *Chloroflexus aurantiacus*, which performs a purple bacterial-type reaction (Achenbach et al. 2001), but in our case *C. aurantiacus* was not detected within the investigated wastewater stabilization ponds.

Another important bacterial sulfur-oxidizing community was identified within the stabilization plant. A total of 15.5% of the retrieved 16S rRNA gene sequences were assignd to the GSB phylum*.* These bacteria carry out anoxygenic photosynthesis with reduced sulfur compounds such as sulfide and elemental sulfur and, for some species, thiosulfate as the electron donor for photoautotrophic growth (Frigaard and Dahl 2009). Some GSB also use hydrogen and ferrous iron as the electron donors. GSB are obligately anaerobic and obligately photoautotrophic, and they form a phylogenetically and physiologically distinct group (Imhoff 2008). They are commonly found in anoxic and sulfide-rich freshwater and estuarine environments as well as in wastewater treatment plants (Siefert et al. 1978) where they may form green or brown bloom depending on their light-harvesting pigments (bacteriochlorophyll *c* or *d* or *e*).

While the ecology of GSB and PSB is to some extent similar (Overmann 2008), their oxidative sulfur metabolism probably shares many characteristics as well (Brune 1995). GSB have a high affinity for sulfide, which is usually the preferred substrate even if other sulfur substrates are available (Brune 1995). The GSB affinity for sulfide is one order of magnitude higher than that of *Chromatiaceae* (Van Gemerden and Mas 1995). In addition, GSB are capable of using significantly lower light intensities for photosynthetic growth. Consequently, green sulfur bacteria thrive below layers of *Chromatiaceae* in close association with the sulfate-reducing bacteria.

### Congruency of phylogenetic results

Congruent phylogenetic results were obtained by applying *apr*A and *dsr*B genes for the characterization of the SRB community with exception concerning *D. tiedjei*. Both functional marker genes allowed the detection of representatives belonging to the *Desulfobulbaceae*, *Desulfobacteraceae*, and *Desulfomicrobiaceae* families. SRB representatives of the *Desulfococcus*/*Desulfosarcina* cluster, *Desulfobacter*, *Desulfobulbus*, and *Desulfomicrobium* were found in both *dsr*B and *apr*A data.

The sulfate-reducing bacterium *D. tiedjei* was only detected by *apr*A-based DGGE approach. This bacterial species is a lateral gene transfer-affected member of the *Syntrophobacteraceae* family, the reclassification of which through *Deltaproteobacteria* is proposed based on the phylogenetic relationship of the xenologous *apr*BA genes (Meyer and Kuever 2007a). While all sequences retrieved with the *dsr* and *apr* primers were dsr and apr sequences, only one of the 16S rRNA sequences was clearly related to sulfate-reducing bacteria; this difference may be attributed to the fact that these functional genes are directly linked to the dissimilatory sulfate reduction and sulfur oxidation processes whereas the ecophysiology and the metabolic function of the microorganisms characterized only by the 16S rRNA sequence remain largely unknown. Furthermore, the phylogenetic specificity of the *dsr*B and *apr*A primer sets was superior to the specificity of the 16S rRNA primer set used herein for revealing the SRB community, while both primers used in this study target all known groups of sulfate-reducing bacteria (Wagner et al. 1998). The differences observed in resolving the diversity of the phototrophic SOB community between *apr*A and *puf*M genes may be attributed to the limited distribution of *apr*A gene among the photosynthetic sulfur-oxidizing bacterial community. So, the use of different genes to characterize microbial communities as has been performed in this study nicely complements the limitations and biases of the different individual genes and will hence give a good overview of the different members that are present in our WSP system.

The data recovered from the 16S rRNA, *dsr*B, *apr*A, and *puf*M genes were complementary. The 16S rRNA genes allowed the determination of the major bacterial groups independently on their physiological or metabolic capacities, while *dsr*B and *apr*A genes were used to get insight on the sulfur metabolism pathways. The *apr*A gene allowed the identification of the SOB and SRB communities concomitantly since the primer used targets the same conserved gene region (Meyer and Kuever 2007c). In contrary, the primer designed on *dsr*B is not specific for SOB analysis and all *dsr*B sequences belonged to SRB. Despite that the core molecular unit *dsr*ABCNMKJOP is common to both sulfur oxidizers and sulfate reducers, only *dsr*EFH and *dsr*L are specific for SOB (Grimm et al. 2008). The *puf*M gene—while not directly involved in sulfur oxidation process—allowed the detection of all anoxygenic purple phototrophic bacteria, most representatives of which are able to oxidize sulfide. The use of functional genes of SOB such as *sox* and *sqr* genes will offer a better view of the sulfide oxidation pathways among this photosynthetic bacterial community.

The PCR–DGGE approach has proven efficient in studying the microbial ecology of wastewater treatment systems as reviewed by Sanz and Köchling (2007). Although the approach provides new insights into the genetic and metabolic composition of these ecosystems, limitations may exist, such as the difficulty in band isolation and the overestimation of sequence heterogeneity in single DGGE bands (Zhang et al. 2005). In addition, the limited DNA sequence information obtained from these relatively short fragments (i.e., *puf*M, 229 bp; *apr*A, 400 bp) can lessen the specificity of the phylogenetic identification. Apart from limitations attributed to DGGE, general biases, such as DNA extraction efficiency, inhibition of PCR, differential amplification, and the incidence of artifact bands due to excessive amplification cycles (Moura et al. 2009), have to be taken into account as well.

### Ecological significance of sulfur bacteria in WSP systems

In such aquatic ecosystems as WSP, a complex microbial consortium with interacting and complementary metabolic processes often exist where major metabolic bacterial groups, such as methanogens, nitrifiers, SRB, sulfide-oxidizing bacteria, and fermenters, can coexist and dominate when the conditions favor their metabolic processes. Based on the 16S rRNA gene database, the dominant metabolism is not linked to sulfate reduction and neither to sulfide oxidation; consequently, fermentation and syntrophic pathways might be important but this cannot be discerned with the 16S rRNA gene results. *Chlorobi* (15.5%) was one of the most dominant bacterial groups identified in this study. Representatives of this phylum share a large set of orthologues to accomplish the demanding task of photosynthesis and sulfur oxidation. They utilize various combinations of sulfide, elemental sulfur, and thiosulfate and sometimes also ferrous iron and hydrogen (Goh et al. 2009).

Since the sulfur cycle involves the presence of SRB and sulfide-oxidizing bacteria, the identification of *Chlorobi*, PSB, and PNSB in the WSP suggest the presence of sulfate-reducing bacteria. This was confirmed by the detection of both *dsr*B and *apr*A sequences related to SRB. Due to their strict anaerobic character, SRB identified herein are likely to dominate within the anoxic and the facultative ponds (i.e., in sediments and the deeper water layers) or within anoxic micro-niches rather than in the maturation ponds where they may constitute only a minor component of bacterial community.

The SRB reduce sulfate to sulfide using either hydrogen as an energy source or CO_2_, acetate, lactate, and other short-chain carboxylic acids as carbon and energy sources. In this study, two SRB communities were distinguished: the SRB with respiratory type of metabolism such as *Desulfobacter*, *Desulfonema*, and *Desulfomonile* and SRB community with both respiratory and fermentative type of metabolism like *Desulfotignum*, *Desulfomicrobium*, and *Desulfobulbus*. The sulfide generated by the SRB activity constitutes a major electron donor for the phototrophic purple sulfur bacteria and may be also used by purple non-sulfur bacteria identified herein such as *Rhodopseudomonas*, *Rhodobacter*, and *Rhodospirillum*. Sulfide oxidation to sulfate would prevent accumulation of sulfide in the wastewater stabilization ponds. The sulfur-oxidizing microbial community within the wastewater stabilization plant is complex and it was suggested to consist of the phototrophic *Gammaproteobacteria* sulfur-oxidizing representatives, the phototrophic *Alphaproteobacteria* and *Betaproteobacteria* SOB (PNSB), and the photoautotrophic sulfur-oxidizing green sulfur bacteria.

The collective data obtained in this study provided insights regarding the composition and the structure of the sulfur microbial community within a wastewater stabilization plant; this may allow a better understanding of the seasonal changes that may affect the microbial community structure especially during the spring and the summer seasons and thus the wastewater treatment’s efficiency. Certainly, in pond design, both effluent characteristics and bacterial community should be taken into account and each pond provides the proper environmental conditions needed for bacterial growth, that is why complex bacterial communities should be monitored in order to guarantee the efficiency of the WSP. The relatively high phylogenetic diversity of the anoxygenic photosynthetic purple and green bacteria reflects their predominance among the total bacterial community within the wastewater stabilization plant since they constitute the most prominent group among the sulfur-oxidizing bacterial community. The diversity of the purple anoxygenic phototrophic bacteria traduces their ecological role in the wastewater treatment process. These bacteria, stimulated by the degradation of environmental parameters during the warm seasons (spring and summer), flourish by forming red-water and may contribute also to biological balance restoration. Further studies on these bacteria can contribute to a better understanding of their roles in these ecosystems.

## Electronic supplementary materials

Below is the link to the electronic supplementary material.

ESM 1 Electronic supplementary material: DGGE analysis of gene fragments from 4 wastewater stabilization ponds. (A) 16S rDNA fragments, (B) dsrB gene fragments, (C) aprA gene fragments, and (D) pufM gene fragments. Lane 1 to 5, sediment samples from the anaerobic pond (A). Lane 6-10, sediment samples from the facultative pond (F). Lane 11, water sample from the anaerobic pond. Lane 12, water sample from the facultative pond. Lanes 13 and 14, water samples from maturation pond M1. Lanes 15 and 16, water samples from maturation pond M2. (JPEG 2.21 kb)

High resolution image (EPS 19700 kb)

## References

[CR1] Abis KL, Mara DD (2006). Temperature measurement and stratification in facultative waste stabilization ponds in the UK climate. Environ Monit Assess.

[CR2] Achenbach LA, Carey J, Madigan MT (2001). Photosynthetic and phylogenetic primers for detection of anoxygenic phototrophs in natural environments. Appl Environ Microbiol.

[CR3] Anceno AJ, Ozaki M, Dang YND, Chuluun B, Shipin OV (2007). Canal networks as extended waste stabilization ponds: fate of pathogens in constructed waterways in Pathumthani province, Thailand. Water Sci Technol.

[CR4] APHA—American Public Health Association (1995). Standard methods for the examination of water and wastewater.

[CR5] Arroyo P, Gemma A, Ivan B, Patricia M, de Estanislao LC, Luis E, de Saenez M (2010). Comparative analysis of the composition of bacterial communities from two constructed wetlands for municipal and swine wastewater treatment. J Water Health.

[CR6] Asano R, Sasaki T, Nakai Y (2007). Isolation and characterization of sulphur oxidizing bacteria from cattle manure compost. Anim Sci J.

[CR7] Baena S, Fardeau ML, Labat M, Ollivier B, Garcia JL, Patel BKC (1998). *Desulfovibrio aminophilus* sp. nov., a novel amino acid degrading and sulphate reducing bacterium from an anaerobic dairy wastewater lagoon. J. Syst Appl Microbiol.

[CR8] Belila A, Gtari M, Ghrabi A, Hassen A (2009). Purple anoxygenic phototrophic bacteria distribution in Tunisian wastewater stabilization plant exhibiting red water phenomenon. Ann Microbiol.

[CR9] Ben-Dov E, Brenner A, Kushmaro A (2007). Quantification of sulfate-reducing bacteria in industrial wastewater, by real-time polymerase chain reaction (PCR) using *dsr*A and *aps*A genes. Microb Ecol.

[CR10] Boon N, De Windt W, Verstraete W, Top EM (2002). Evaluation of nested PCR–DGGE (denaturing gradient gel electrophoresis) with group-specific 16S rDNA primers for the analysis of bacterial communities from different wastewater treatment plants. FEMS Microbiol Ecol.

[CR11] Brune DC, Blankenship RE, Madigan MT, Bauer CE (1995). Sulfur compounds as photosynthetic electron donors. Anoxygenic photosynthetic bacteria.

[CR12] Corson GE, Nagashima KVP, Matsuura K, Sakuragi Y, Wettasinghe R, Qin H, Allen R, Knaff DB (1999). Genes encoding light harvesting and reaction center proteins from *Chromatium vinosum*. Photosynth Res.

[CR13] Cravo-Laureau C, Matheron R, Cayol J-L, Joulian C, Hirschler-Réa A (2004). *Desulfatibacillum aliphaticivorans* gen. nov., sp. nov., an n-alkane- and n-alkene-degrading sulfate-reducing bacterium. Int J Syst Evol Microbiol.

[CR14] Curtis TP, Mara DD, Dixo NGH, Silva SA (1994). Light penetration in waste stabilization ponds. Water Res.

[CR15] Dahl C, Prange A, Shively JM (2006). Bacterial sulfur globules: occurrence, structure, and metabolism. Bacterial inclusions (microbiological monographs (1)).

[CR16] Dar SA, Kuenen JG, Muyzer G (2005). Nested PCR denaturing gradient gel electrophoresis approach to determine the diversity of sulfate-reducing bacteria in complex microbial communities. Appl Environ Microbiol.

[CR17] Dar SA, Yao L, Van Dongen U, Kuenen JG, Muyzer G (2007). Analysis of diversity and activity of sulfate reducing bacterial communities in sulfidogenic bioreactors using 16S rRNA and *dsr*B genes as molecular markers. Appl Environ Microbiol.

[CR18] Devereux R, Delaney M, Widdel F, Stahl DA (1989). Natural relationships among sulfate-reducing eubacteria. J Bacteriol.

[CR19] Ding L, Zhou Q, Wang L, Zhang Q (2010). Dynamics of bacterial community structure in a fullscale wastewater treatment plant with anoxic–oxic configuration using 16S rDNA PCR–DGGE fingerprints. Afr J Biotechnol.

[CR20] Frigaard NU, Dahl C (2009). Sulfur metabolism in phototrophic sulfur bacteria. Adv Microb Physiol.

[CR21] Geets J, Borremans B, Diels L, Springael D, Vangronsveld J, Van der Lelied D, Vanbroekhoven K (2006). *Dsr*B gene-based DGGE for community and diversity surveys of sulfate-reducing bacteria. J Microbiol Meth.

[CR22] Ghosh W, Dam B (2009). Biochemistry and molecular biology of lithotrophic sulfur oxidation by taxonomically and ecologically diverse bacteria and Archaea. FEMS Microbiol Rev.

[CR23] Goh SHM, Mabbett AN, Welch JP, Hall SJ, McEwan AG (2009). Molecular ecology of a facultative swine waste lagoon. Lett Appl Microbiol.

[CR24] Grimm F, Franz B, Dahl C (2008). Thiosulfate and sulfur oxidation in purple sulfur bacteria. Microbial sulfur metabolism.

[CR25] Imhoff JF (2005) The anoxygenic phototrophic purple bacteria. In: Boone, Castenholz and Garrity (ed), Bergey’s Manual of Systematic Bacteriology, 2nd edn,Vol 1, Springer-Verlag, New York, pp 631-637

[CR26] Imhoff JF, Hell R, Dahl C, Knaff DB, Leustek T (2008). Systematics of anoxygenic phototrophic bacteria. Sulfur metabolism in phototrophic organisms.

[CR27] Karr EA, Sattley WM, Jung DO, Madigan MT, Achenbach LA (2003). Remarkable diversity of phototrophic purple bacteria in a permanently frozen Antarctic lake. Appl Environ Microbiol.

[CR28] Konig A, Pearson HW, Silva SA (1987). Ammonia toxicity to algal growth in waste stabilization ponds. Water Sci Technol.

[CR29] Kopriva S, Patron N, Leustek T, Keeling P, Hell R, Leustek T, Dahl C, Knaff D (2008). Phylogenetic analysis of sulfate assimilation and cysteine biosynthesis in phototrophic organisms. Advances in photosynthesis and respiration.

[CR30] Kubo K, Knittel K, Amann R, Fukui M, Matsuura K (2011). Sulfur-metabolizing bacterial populations in microbial mats of the Nakabusa hot spring. Syst Appl Microbiol.

[CR31] Lai PCC, Lam PKS (1997). Major pathways for nitrogen removal in waste water stabilization ponds. Water Air Soil Pollut.

[CR32] LaPara TM, Nakatsu CH, Pantea L, Allemann JE (2000). Phylogenetic analysis of bacterial communities in mesophilic and thermophilic bioreactors treating pharmaceutical wastewater. Appl Environ Microbiol.

[CR33] Lücker S, Steger D, Kjeldsen KU, MacGregor BJ, Wagner M, Loy A (2007). Improved 16S rRNA targeted probe set for analysis of sulfate-reducing bacteria by fluorescence in situ hybridization. J Microbiol Meth.

[CR34] Ludwig W, Strunk O, Westram R, Richter L, Meier H, Yadhukumar A, Buchner T, Lai S, Steppi G, Jobb W, Förster I, Brettske S, Gerber AW, Ginhart O, Gross S, Grumann S, Hermann R, Jost A, König T, Liss R, Lüssman M, May B, Nonhoff B, Reichel R, Strehlow A, Stamatakis N, Stuckmann A, Vilbig M, Lenke T, Ludwig AB, Schleifer K-H (2004). ARB: a software environment for sequence data. Nucleic Acids Res.

[CR35] Madigan MT, Blankenship RE, Madigan MT, Bauer CE (1995). Microbiology of nitrogen fixation in photosynthetic bacteria. Anoxygenic photosynthetic bacteria.

[CR36] Madigan MT, Martinko JM (2006). Brock biology of microorganisms.

[CR37] Malik S, Beer M, Megharaj M, Naidu R (2008). The use of molecular techniques to characterize the microbial communities in contaminated soil and water. Environ Int.

[CR38] Mara DD, Al Baz I, Otterpohl R, Wendland C (2008). Waste stabilization ponds: a highly appropriate wastewater treatment technology for Mediterranean countries. Efficient management of wastewater: its treatment and reuse in water-scarce countries.

[CR39] Meyer B, Kuever J (2007). Phylogeny of the alpha and beta subunits of the dissimilatory adenosine-5′-phosphosulfate (APS) reductase from sulfate-reducing prokaryotes—origin and evolution of the dissimilatory sulfate-reduction pathway. Microbiology.

[CR40] Meyer B, Kuever J (2007). Molecular analysis of the distribution and phylogeny of dissimilatory adenosine-5′-phosphosulfate reductase-encoding genes (*aprAB*) among sulfur-oxidizing prokaryotes. Microbiology.

[CR41] Meyer B, Kuever J (2007). Molecular analysis of the diversity of sulfate-reducing and sulfur-oxidizing prokaryotes in the environment, using *apr*A as functional marker gene. Appl Environ Microbiol.

[CR42] Meyer B, Kuever J (2008). Phylogenetic diversity and spatial distribution of the microbial community associated with the Caribbean deep-water sponge *Polymastia* cf*. corticata* by 16S rRNA*, apr*A, and *amo*A gene analysis. Microb Ecol.

[CR43] Meyer B, Imhoff JF, Kuever J (2007). Molecular analysis of the distribution and phylogeny of the *soxB* gene among sulphur-oxidizing bacteria—evolution of the Sox sulphur oxidation enzyme system. Environ Microbiol.

[CR44] Miletto M, Bodelier PLE, Laanbroek HJ (2007). Improved PCR–DGGE for high resolution diversity screening of complex sulfate-reducing prokaryotic communities in soil and sediments. J Microbiol Meth.

[CR45] Mohanakrishnan J, Kofoed MV, Barr J, Yuan Z, Schramm A, Meyer RL (2011). Dynamic microbial response of sulfidogenic wastewater biofilm to nitrate. Appl Microbiol Biotechnol.

[CR46] Moura A, Tacão M, Henriques I, Dias J, Ferreira P, Correia A (2009). Characterization of bacterial diversity in two aerated lagoons of a wastewater treatment plant using PCR–DGGE analysis. Microbiol Res.

[CR47] Muyzer G, Stams AJM (2008). The ecology and biotechnology of sulphate-reducing bacteria. Nat Rev Microbiol.

[CR48] Muyzer G, Teske A, Wirsen CO, Jannasch HW (1995). Phylogenetic relationships of *Thiomicrospira* species and their identification in deep-sea hydrothermal vent samples by denaturing gradient gel electrophoresis of 16S rDNA fragments. Arch Microbiol.

[CR49] Nair C (1992). Pollution control through water conservation and wastewater reuse in the fish processing industry. Water Sci Technol.

[CR50] Okabe S, Itoh T, Satoh H, Watanabe Y (1999). Analyses of spatial distributions of sulfate-reducing bacteria and their activity in aerobic wastewater biofilms. Appl Environ Microbiol.

[CR51] ONAS (2009). Annual report of the National Office of Sanitation.

[CR52] Oude Elferink SJWH, Visser A, Hulshoff PLW, Stams AJM (1994). Sulfate reduction in methanogenic bioreactors. FEMS Microbiol Rev.

[CR53] Overmann J, Hell R, Dahl C, Knaff DB, Leustek T (2008). Ecology of phototrophic sulfur bacteria. Advances in photosynthesis and respiration.

[CR54] Pearson HW (1986). Estimation of chlorophyll *a* as a measure of algal biomass in waste stabilization ponds.

[CR55] Pierson BK, Olson JM, Amesz J (1987). Photosynthetic bacteria. Photosynthesis.

[CR56] Rabus R, Hansen TA, Widdel F, Dworkin M, Falkow S, Rosenberg E, Schleifer KH, Stackebrandt E (2006). Dissimilatory sulfate and sulfur-reducing. The prokaryotes, vol. 2.

[CR57] Ranchou-Peyruse A, Herbert R, Caumette P, Guyoneaud R (2006). Comparison of cultivation dependent and molecular methods for studying the diversity of anoxygenic purple phototrophs in sediments of a eutrophic brackish lagoon. Environ Microbiol.

[CR58] Raskin L, Zheng D, Griffin ME, Stroot PG, Misra P (1995). Characterization of microbial communities in anaerobic bioreactors using molecular probes. Antonie Van Leeuwenhoek.

[CR59] Reinoso R, Bécares E (2008). Environmental inactivation of *Cryptosporidium parvum* oocysts in waste stabilization ponds. Microb Ecol.

[CR60] Sander J, Dahl C, Hunter CN, Daldal F, Thurnauer MC, Beatty JT (2008). Metabolism of inorganic sulfur compounds in purple bacteria. The purple phototrophic bacteria.

[CR61] Sanz JL, Köchling T (2007). Molecular biology techniques used in wastewater treatment: an overview. Process Biochem.

[CR62] Schäfer H, Muyzer G (2001) Denaturing gradient gel electrophoresis in marine microbial ecology. In: Paul J (ed) Methods in Microbiology, Vol 30, Academic Press, London, pp 425–468

[CR63] Shammas NK, Wang LK, Wu Z, Wang LK, Pereira NC, Hung YT (2009). Wastewater stabilization ponds and lagoons. Biological treatment processes. Biological treatment processes.

[CR64] Shimada K, Blankenship RE, Madigan MT, Bauer CE (1995). Aerobic anoxygenic phototrophs. Anoxygenic photosynthetic bacteria.

[CR65] Siefert E, Irgens RL, Pfennig N (1978). Phototrophic purple and green bacteria in a sewage treatment plant. Appl Environ Microbiol.

[CR66] Tang K, Baskaran V, Nemati M (2008). Bacteria of the sulphur cycle: an overview of microbiology, biokinetics and their role in petroleum and mining industries. Biochem Eng J.

[CR67] Tank M, Thiel V, Imhoff JF (2009). Phylogenetic relationship of phototrophic purple sulfur bacteria according to *puf*L and *puf*M genes. Int Microbiol.

[CR68] Tyagi VK, Kazmi AA, Chopra AK (2008). Removal of fecal indicators and pathogens in a waste stabilization pond system treating municipal wastewater in India. Water Environ Res.

[CR69] Van Gemerden H, Mas J, Blankenship RE, Madigan MT, Bauer CE (1995). Ecology of phototrophic sulfur bacteria. Anoxygenic photosynthetic bacteria. Advances in photosynthesis, vol 2.

[CR70] Vannini C, Munz G, Mori G, Lubello C, Verni F, Petroni G (2008). Sulphide oxidation to elemental sulfur in a membrane bioreactor: performance and characterization of the selected microbial sulphur-oxidizing community. Syst Appl Microbiol.

[CR71] Veenstra S, Al-Nozaily FA, Alaerts GJ (1995). Purple non-sulfur bacteria and their influence on waste stabilization pond performance in the Yemen Republic. Water Sci Technol.

[CR72] Villanueva J, Grimalt JO, Wit RD, Brendan JK, Maxwell JR (1994). Sources and transformations of chlorophylls and carotenoids in a monomictic sulphate-rich karstic lake environment. Org Geochem.

[CR73] Wagner M, Loy A (2002). Bacterial community composition and function in sewage treatment systems. Curr Opin Biotechnol.

[CR74] Wagner M, Amann R, Lemmer H, Schleifer KH (1993). Probing activated sludge with oligonucleotides specific for proteobacteria: inadequacy of culture-dependent methods for describing microbial community structure. Appl Environ Microbiol.

[CR75] Wagner M, Roger AJ, Flax JL, Brusseau GA, Stahl DA (1998). Phylogeny of dissimilatory sulfite reductases supports an early origin of sulfate respiration. J Bacteriol.

[CR76] Wagner A, Loy R, Nogueira U, Purkhold LN, Daims H (2002). Microbial community composition and function in wastewater treatment plants. Antonie Van Leeuwenhoek.

[CR77] Widdel F, Zehnder AJB (1988). Microbiology and ecology of sulphate and sulphur reducing bacteria. Biology of anaerobic microorganisms.

[CR78] Xia S, Duan L, Song Y, Li J, Piceno YM, Andersen GL, Alvarez-Cohen L, Moreno-Andrade I, Huang CL, Hermanowicz SW (2010). Bacterial community structure in geographically distributed biological wastewater treatment reactors. Environ Sci Technol.

[CR79] Zhang X, Yan X, Gao P, Wang L, Zhou Z, Zhao L (2005). Optimized sequence retrieval from single bands of temperature gradient gel electrophoresis profiles of the amplified 16S rDNA fragments from an activated sludge system. J Microbiol Methods.

