# Supplementary figures and images for: Sulfur bacteria in wastewater stabilization ponds periodically affected by the ‘red-water’ phenomenon
Source: Appl Microbiol Biotechnol. 2012 Feb 23;97(1):379–94. doi: 10.1007/s00253-012-3931-5 (PMC3536956; doi:10.1007/s00253-012-3931-5)

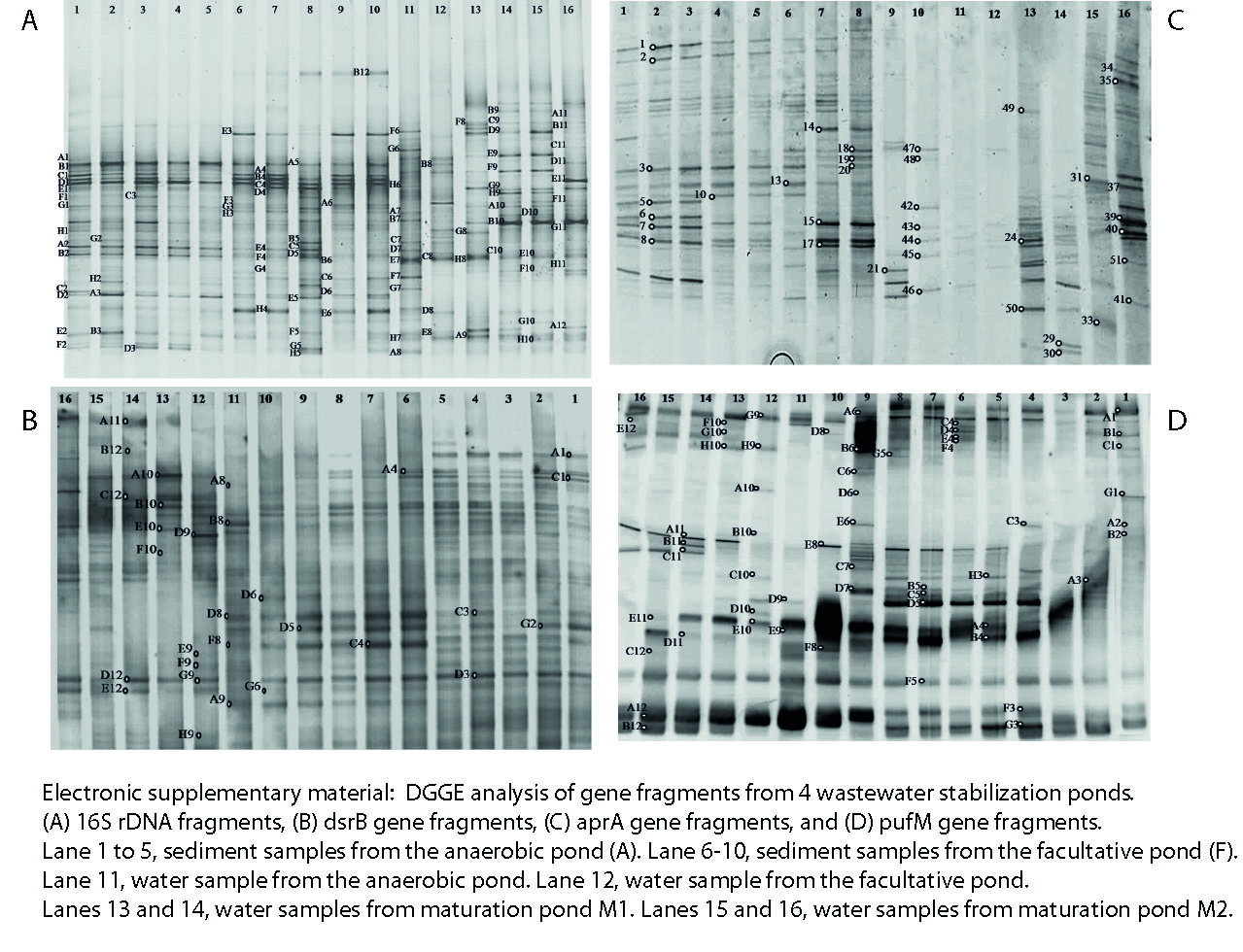

Supplement: Supplementary file 1 — Electronic supplementary material: DGGE analysis of gene fragments from 4 wastewater stabilization ponds. (A) 16S rDNA fragments, (B) dsrB gene fragments, (C) aprA gene fragments, and (D) pufM gene fragments. Lane 1 to 5, sediment samples from the anaerobic pond (A). Lane 6-10, sediment samples from the facultative pond (F). Lane 11, water sample from the anaerobic pond. Lane 12, water sample from the facultative pond. Lanes 13 and 14, water samples from maturation pond M1. Lanes 15 and 16, water samples from maturation pond M2. (JPEG 2.21 kb) [file 253_2012_3931_Fig2_ESM.jpg]
